# Twenty-Three Years at Bethlem Hospital

**Published:** 1911-08-12

**Authors:** 


### TWENTY-THREE YEARS AT BETHLEM HOSPITAL.

The recent retirement of Dr. Theo. Hyslop from the post of Medical Superintendent of the Bethlem Royal Hospital marks an important phase in the treatment of the insane in this country. That phase has been illustrated in the change that has come over public speech in regard to the nursing of insane persons. The asylum attendant of the original days, when an insane person was regarded as a lunatic—that is to say, as a moonstruck or accursed person—has become the mental nurse of a mentally diseased patient. The word asylum, which had become a public horror, as recognised in fiction in such popular novels as "The Woman in White," has been exchanged for the reassuring term "mental hospital." The cell with its litter of straw is a thing of the past, and the humane treatment of mental cases has been accompanied by a definite interest in, and attention to, their psychology. The association frequently alleged by such writers as Lombroso of insanity and genius has produced a new scientific interest in the problem of the lunatic, and psychological treatment is now recognised as an important part of mental nursing and management. It becomes interesting, therefore, to gather from Dr. Hyslop's own experience of twenty-three years at the Bethlem Royal Hospital some of the changes which he has seen, and which have accompanied, whether as cause or effect, this change in public opinion.

### Early Years at Bethlem.

The first ten years of Dr. Hyslop's experience were under Dr. Savage and Dr. Percy Smith. For the last thirteen of them he was himself in charge. During his period of superintendence the last traces of restraint were removed, and the old hospital, which as a foundation had known more than one change of site, was on the latest of them, in Lambeth, in need of some reconstruction. One of the more important factors in this work was the installation of a completely renewed drainage system. This was a more difficult problem for the administrators than at first sight might be thought. It necessitated the closing of the hospital for several months, and it involved the finding of temporary homes for the patients, a class obviously for whom at no time it is easy to cater. The staff itself had to move elsewhere.

Hospital managers will appreciate the multifarious administrative details which required attention and supervision at this time, details which often involved legal points of difficult interpretation, apart from the difficulties inherent in any attempt to administer patients and staff who were housed in so many places. The great business, however, at length was accomplished in 1904.

### Reconstruction.

It will be seen that the hospital buildings required alteration before it was possible to undertake in any elaboration the psychological treatment of its inmates. The spade work had to be started first. Thus the relics of an older time survived in the undressed walls that were still to be found in many of the rooms. The work of plastering them was incomplete, and it was only when this had been finished that the question of their decoration could be considered, a question which, as affecting the environment of the patients, was to be most carefully pondered later on.

Another point, which was more obvious to public gaze, was the type of window which prevailed in the hospital, a type that was more suggestive of a prison than of an institution for the care of the diseased. The whole place was prison-like in appearance; light and air were largely excluded from the rooms, with the result that these became dreary and punitive in aspect, and from the point of view of the modern hospital architect were ill ventilated and lighted. The structure of the hospital buildings was thus interfered with; walls were knocked down, apertures made, and light allowed to enter more freely. By such measures was the environment of the patients improved.

### Improving the Hospital.

Up-to-date organisation now became necessary, and a system of fire-prevention was introduced, with suitable training and practised drills. Electric light was installed, and a telephone system, which has been found useful in the administration for several reasons. The elaboration of the pathological and physiological laboratories was undertaken also, and the gardens were rearranged. This rearrangement took the form of the removal of the gloomy bushes which lent the grounds at that time a somewhat funereal air. Colour replaced shadow, and flowers were planted in numbers about the place. Externally, therefore, in the buildings and in the gardens, the aim was to produce an atmosphere of light and cheerfulness.

This was preparatory to an important change in the treatment of the "inmates," who had now become "patients" worthy of solicitude.

### The Symphony Concerts.

Probably the chief fact that will be remembered concerning the superintendence of Dr. Hyslop is the encouragement which he gave to the practice and enjoyment by the patients of the fine arts. Every form of pleasurable activity likely to improve the condition of the patients and to keep them happy and contented was looked upon with approval by him. For example, a symphony orchestra was established in which the patients took part, and the works of Mozart and Beethoven, Gluck and Verdi, were performed to large audiences. It is interesting to think of the Bethlem patients as not deprived of the Ninth Symphony, or as listening with enjoyment to the delightful dance music in "Armide," or to the songs in "Don Giovanni." Selections from operas were included at these concerts, the programmes for which sometimes contained scores composed by the medical superintendent himself. This fact is worth mentioning, as it gives a valuable emphasis to the enthusiasm with which, as we have stated, the humanities were pursued. These concerts became a regular feature of the hospital's life, and Dr. Hyslop is understood to have found that the patients responded more easily to the stimulus of music, where of course form and content are indissolubly joined in one appeal to the senses, than to that of colour or rhythm, where the influence of other elements, the disturbing factor of the intellect among them, destroys the unity which music possesses—as its most obvious character.

The other arts, however, were not neglected, and painting was encouraged, with the result that an exhibition of 502 pictures painted by the patients was held in 1901.

That fact is enough to show that this policy was popular, and apart from the interest to the patients that the practice of painting afforded, the student of art finds much to interest him in the attempts of lunatics to express their visions in colour or line. Common faults of drawing are often traceable to particular optical defects, but the insane patient's work possesses not infrequently a quality of freshness, of unspoiled imagination, that is as welcome as the conceptual drawings of a child, in which the lack of perspective, for instance, is sometimes compensated by an imaginative vigour that is lost later when accurate observation of the object drawn has become regarded as of overwhelming importance.

It has been impossible to speak otherwise than in very general terms of the changes which have taken place at Bethlem in the twenty-three years of Dr. Hyslop's connection with it. But his superintendence has coincided with a change of view in the attitude of those in charge of lunatics which is both humane and interesting. Only by its development can the psychology of these unfortunates be observed sufficiently for inductions to be made from it, and the success which attended Dr. Hyslop's efforts in educing latent humanities in them shows the value and scope for the future in their continually widened activity. Like the lepers, but perhaps later, the insane have become recognised as diseased but not inhuman beings. One side of our public recognition of this is illustrated in the changes that have taken place at Bethlem.

